# Supplementary material for: The lived experience of long COVID: A qualitative study of mental health, quality of life, and coping
Source: PLoS One. 2023 Oct 13;18(10):e0292630. doi: 10.1371/journal.pone.0292630 (PMC10575511; doi:10.1371/journal.pone.0292630)
Supplement: Attachment — Submitted filename: The lived experience of long COVID.docx [file pone.0292630.s001.docx]

**The lived experience of long COVID**

**Comments to the authors**

Thank you for the opportunity to review this manuscript, reporting a qualitative study of the lived experience of long COVID. It really is a beautifully written piece, and I have no requests for any major amendments. The study design, including recruitment, ethics, data collection and analysis is well described, and it is refreshing to see open acknowledgement of the contribution of the advisory group to the study. The findings are impactful and shine a spotlight on the plight of Canadians living and struggling with the consequences of long COVID. The only tiny little suggestions I would make would be:

1. To present the theme labels in italics in the text wherever these are first mentioned and capitalise the first word of each theme, and
2. to avoid the word ‘include ‘ since it suggests there are other sub-themes that are not being mentioned.

For example, on p12: **Theme 1: The emotional Landscape of long COVID**. At the end of this introductory paragraph, you write: ‘Subthemes which represent this experience include: anxiety and fear of the unknown, challenges with emotional regulation, and feelings of hopelessness and depression’. That is quite tricky for the reader to unpick, but if you did this (and applied it throughout wherever appropriate), you would make things much easier for the reader:

‘Three subthemes represent this experience: *Anxiety and fear of the unknown*, *Challenges with emotional regulation*, and *Feelings of hopelessness and depression*’ …

Thank you again – it has been a pleasure to review this work.
