# Supplementary material for: The lived experience of long COVID: A qualitative study of mental health, quality of life, and coping
Source: PLoS One. 2023 Oct 13;18(10):e0292630. doi: 10.1371/journal.pone.0292630 (PMC10575511; doi:10.1371/journal.pone.0292630)
Supplement: Attachment — Submitted filename: Reviewer comments.docx [file pone.0292630.s002.docx]

Journal Requirements:

When submitting your revision, we need you to address these additional requirements.

1. Please ensure that your manuscript meets PLOS ONE's style requirements, including those for file naming. The PLOS ONE style templates can be found at 
[https://journals.plos.org/plosone/s/file?id=wjVg/PLOSOne_formatting_sample_main_body.pdf](https://urldefense.com/v3/__https:/journals.plos.org/plosone/s/file?id=wjVg*PLOSOne_formatting_sample_main_body.pdf__;Lw!!FxkXuJIC!blulJ6AgjaIwBntVBzG2-6sgGWR27Tuq0tcb2W58uaWkK-oX3oOv6v4R_07FLKq3wlDcXIYlWWeiqIUa8Q$) and 
[https://journals.plos.org/plosone/s/file?id=ba62/PLOSOne_formatting_sample_title_authors_affiliations.pdf](https://urldefense.com/v3/__https:/journals.plos.org/plosone/s/file?id=ba62*PLOSOne_formatting_sample_title_authors_affiliations.pdf__;Lw!!FxkXuJIC!blulJ6AgjaIwBntVBzG2-6sgGWR27Tuq0tcb2W58uaWkK-oX3oOv6v4R_07FLKq3wlDcXIYlWWfMB2Fcjw$)

- We have reviewed this for consistency.

2. We note that the grant information you provided in the ‘Funding Information’ and ‘Financial Disclosure’ sections do not match.

- Please note that the financial disclosure is not the funding that was provided for this study, but is rather a separate disclosure that that co-author makes. We have added the funding information to that statement as well for clarity.

When you resubmit, please ensure that you provide the correct grant numbers for the awards you received for your study in the ‘Funding Information’ section.

- We have done so in the system.

3. Thank you for stating the following in the Competing Interests section: 
"David Castle has received grant monies for research from Servier, Boehringer Ingelheim; Travel Support and Honoraria for Talks and Consultancy from Servier, Seqirus, Lundbeck. He is a founder of the Optimal Health Program (OHP), and holds 50% of the IP for OHP; and is part owner of Clarity Healthcare. He does not knowingly have stocks or shares in any pharmaceutical company. Other authors have no conflict of interest to declare."

Please confirm that this does not alter your adherence to all PLOS ONE policies on sharing data and materials, by including the following statement: "This does not alter our adherence to  PLOS ONE policies on sharing data and materials.” (as detailed online in our guide for authors [http://journals.plos.org/plosone/s/competing-interests).](https://urldefense.com/v3/__http:/journals.plos.org/plosone/s/competing-interests).__;!!FxkXuJIC!blulJ6AgjaIwBntVBzG2-6sgGWR27Tuq0tcb2W58uaWkK-oX3oOv6v4R_07FLKq3wlDcXIYlWWexdSc9jw$)  If there are restrictions on sharing of data and/or materials, please state these. Please note that we cannot proceed with consideration of your article until this information has been declared.

Please include your updated Competing Interests statement in your cover letter; we will change the online submission form on your behalf.

We have made this change and put it in the cover letter as requested.

4. We note that you have indicated that data from this study are available upon request. PLOS only allows data to be available upon request if there are legal or ethical restrictions on sharing data publicly. For more information on unacceptable data access restrictions, please see [http://journals.plos.org/plosone/s/data-availability#loc-unacceptable-data-access-restrictions.](https://urldefense.com/v3/__http:/journals.plos.org/plosone/s/data-availability*loc-unacceptable-data-access-restrictions.__;Iw!!FxkXuJIC!blulJ6AgjaIwBntVBzG2-6sgGWR27Tuq0tcb2W58uaWkK-oX3oOv6v4R_07FLKq3wlDcXIYlWWdnNDW17A$)

In your revised cover letter, please address the following prompts:

a) If there are ethical or legal restrictions on sharing a de-identified data set, please explain them in detail (e.g., data contain potentially sensitive information, data are owned by a third-party organization, etc.) and who has imposed them (e.g., an ethics committee). Please also provide contact information for a data access committee, ethics committee, or other institutional body to which data requests may be sent.

Please note that qualitative data contains personal information and is guided by the regulations of the Research Ethics Board of the Centre for Addiction and Mental Health. This study was not originally conceptualized for data sharing, and specific the Research Ethics Board approval would therefore be required. We have provided the contact information. Research Ethics <Research.Ethics@camh.ca>

b) If there are no restrictions, please upload the minimal anonymized data set necessary to replicate your study findings as either Supporting Information files or to a stable, public repository and provide us with the relevant URLs, DOIs, or accession numbers. For a list of acceptable repositories, please see [http://journals.plos.org/plosone/s/data-availability#loc-recommended-repositories](https://urldefense.com/v3/__http:/journals.plos.org/plosone/s/data-availability*loc-recommended-repositories__;Iw!!FxkXuJIC!blulJ6AgjaIwBntVBzG2-6sgGWR27Tuq0tcb2W58uaWkK-oX3oOv6v4R_07FLKq3wlDcXIYlWWet4fM53g$).

We will update your Data Availability statement on your behalf to reflect the information you provide.

5. Please include your full ethics statement in the ‘Methods’ section of your manuscript file. In your statement, please include the full name of the IRB or ethics committee who approved or waived your study, as well as whether or not you obtained informed written or verbal consent. If consent was waived for your study, please include this information in your statement as well.

This has been added.

6. Please review your reference list to ensure that it is complete and correct. If you have cited papers that have been retracted, please include the rationale for doing so in the manuscript text, or remove these references and replace them with relevant current references. Any changes to the reference list should be mentioned in the rebuttal letter that accompanies your revised manuscript. If you need to cite a retracted article, indicate the article’s retracted status in the References list and also include a citation and full reference for the retraction notice.

References have been reviewed in detail.

Additional Editor Comments:

Thank you for submitting this very nice paper which we all enjoyed reading. There are a few minor revisions that would improve the paper please correct any typos and consider the suggestions. I look forward to receiving your revisions.

We thank the editor for their positive appraisal of the manuscript. We have made all of the requested changes. We look forward to your response.

5. Review Comments to the Author

Reviewer #1: Please see the attached file. I enjoyted reading your well-written paper. My comments are brief, buit I wanted to be able to use bold and italics to demonstrate a suggestion I have to you, and could not do that through the online form.

We thank the reviewer. Please see below.

Reviewer #2: This is a very well written paper of a well executed study which will make a valuable contribution to the literature. It needs a good read through for small grammar and tense issues in places --
For example p 3 end of last para—of cough not just cough

Tense p9 above research team positionality heading--- included not include

plural Participants bottom page 12

We thank the reviewer for these comments. We have proofed thoroughly and corrected the items identified.

**The lived experience of long COVID**

**Comments to the authors**

Thank you for the opportunity to review this manuscript, reporting a qualitative study of the lived experience of long COVID. It really is a beautifully written piece, and I have no requests for any major amendments. The study design, including recruitment, ethics, data collection and analysis is well described, and it is refreshing to see open acknowledgement of the contribution of the advisory group to the study. The findings are impactful and shine a spotlight on the plight of Canadians living and struggling with the consequences of long COVID.

We thank the reviewer for the review of our manuscript and the positive comments. We have responded to the comments below and in text.

The only tiny little suggestions I would make would be:

1. To present the theme labels in italics in the text wherever these are first mentioned and capitalise the first word of each theme, and

We have made this revision throughout.

2. to avoid the word ‘include ‘ since it suggests there are other sub-themes that are not being mentioned.

We have clarified each such statement.

For example, on p12: **Theme 1: The emotional Landscape of long COVID**. At the end of this introductory paragraph, you write: ‘Subthemes which represent this experience include: anxiety and fear of the unknown, challenges with emotional regulation, and feelings of hopelessness and depression’. That is quite tricky for the reader to unpick, but if you did this (and applied it throughout wherever appropriate), you would make things much easier for the reader:

‘Three subthemes represent this experience: *Anxiety and fear of the unknown*, *Challenges with emotional regulation*, and *Feelings of hopelessness and depression*’ …

Thank you again – it has been a pleasure to review this work.

We thank the reviewer for illustrating their request and have made these changes.
